# The impact of topical capsaicin application on the muscle metaboreflex and microvascular responsiveness

**DOI:** 10.14814/phy2.70496

**Published:** 2025-08-09

**Authors:** Nikolaus D. Carpenter, Aaron E. Wahl, Alec L. E. Butenas, Sherwin Toribio, Jacob T. Caldwell

**Affiliations:** ^1^ Exercise and Sport Science Department La Crosse Wisconsin USA; ^2^ Breathing Research and Therapeutics Center, Department of Physical Therapy University of Florida Gainesville Florida USA

**Keywords:** capsaicin, metaboreflex, microvascular, near‐infrared spectroscopy

## Abstract

Topical capsaicin reduces metaboreflex responses during post‐exercise circulatory arrest (PECA) in males, but its effects in females and on microvascular responsiveness in either sex remain unclear. This study hypothesized that capsaicin would reduce blood pressure during PECA in males but not females, and enhance microvascular responsiveness in both sexes. Healthy males (*n* = 9) and females (*n* = 11) were randomly assigned to one of three conditions: capsaicin applied to the exercising arm, capsaicin applied to the non‐exercising arm, or no capsaicin (control). Blood pressure was measured via the brachial artery during a handgrip exercise at 30% maximal voluntary contraction, followed by PECA using a rapid inflation cuff. Microvascular responsiveness was assessed with near‐infrared spectroscopy (NIRS) during a post‐occlusive reactive hyperemia test, conducted before and twice after capsaicin application. Capsaicin did not significantly alter blood pressure during PECA (*p* > 0.05), but a significant interaction was observed for microvascular responsiveness (*p* = 0.01). Although topical capsaicin did not attenuate metaboreflex‐induced blood pressure changes, it enhanced microvascular responsiveness in the exercising arm compared to the systemic control. These findings suggest localized vascular benefits of capsaicin, but no clear impact on the metaboreflex. Further research is needed to clarify sex‐specific responses to topical capsaicin.

## 1 INTRODUCTION

Increasing blood pressure during physical activity is a normal and essential physiological response; however, an exaggerated blood pressure increase can elevate the risk of cardiovascular disease and adverse cardiac events (Berger et al., 2015; Laukkanen et al., 2006; Singh et al., 1999). The exercise pressor reflex (EPR) is a peripheral feedback mechanism and consists of type III and IV afferents to adjust the blood pressure and cardiovascular responses during physical activity. Specifically, these reflexes are polymodal and sense mechanical and metabolic changes in the periphery (Greaney et al., 2014; Kaufman & Hayes, 2002). Current evidence highlights the metaboreflex as an essential driver of exaggerated blood pressure responses that elicit pressor responses during both dynamic and isometric muscular contraction (Kaufman & Hayes, 2002). Yet, limited work investigating non‐pharmacological approaches to reduce blood pressure is currently available.

Investigations of the EPR often fail to include both sexes despite the well‐documented differences in blood pressure responses to activity (Smith et al., 2019). For example, pre‐menopausal females demonstrate a blunted metaboreflex to both isometric and rhythmic contraction relative to post‐menopausal females (Gonzales et al., 2007; Hunter & Enoka, 2001; Limberg et al., 2010; Ogawa et al., 1992; Parker et al., 2007). The blunted metaboreflex response may be driven by changes in sex hormones and the transduction of impulses driving pressor responses; however, our understanding of sex hormone phases on the EPR in pre‐menopausal women is lacking.

Capsaicin, the pungent spice component of chili peppers, is known to attenuate the metaboreflex when applied topically in animals (Ichiyama et al., 2002; LeDoux & Wilson, 2001; Nelson et al., 2004) and humans (Dawson et al., 2004; Vianna et al., 2018). Using capsaicin, isolated metaboreflex attenuation in young males was achieved by Vianna et al., in 2018. Currently, little is known about the sex‐specific effects of topical capsaicin on the EPR. Capsaicin and its receptor transient receptor potential vanilloid‐1 (TRPV‐1), expressed in various peripheral and central tissues (Yang et al., 2010), are proposed to act on the EPR through the depletion of secondary messengers, such as neuropeptides like substance P and calcitonin gene‐related peptide, which are necessary for the transmission of nerve signals (Dray, 1992). While this cascade is necessary for afferent transmission of stimuli to cardiovascular control centers, reducing high levels of feedback from the metaboreflex by limiting the reflexive efferent sympathetic drive and attenuating any exaggerated blood pressure response to physical activity would be a valuable finding.

Microvascular dysfunction coexists with hypertensive blood pressure responses and reductions in bioavailable nitric oxide (Houben et al., 2017). Microvascular changes can be assessed using near‐infrared spectroscopy (NIRS), which estimates relative tissue oxygenation through near‐infrared light and correlates with the macrovasculature (Barstow, 2019; McLay, Fontana, et al., 2016; McLay, Nederveen, et al., 2016). Capsaicin activates TRPV‐1 channels to enhance nitric oxide release, promote vasodilation, and reduce the development of atherosclerotic plaque formation (Houben et al., 2017; Randhawa & Jaggi, 2017). Moreover, skeletal muscle arterioles express TRPV‐1 channels that actively oppose sympathetic drive (Ives et al., 2017). While the role of these receptors has been established, the time course and effect of topical capsaicin on noninvasive microvascular responsiveness are unknown.

The purpose of this study was to determine the effect of topical capsaicin cream on the blood pressure and microvascular responses to rhythmic handgrip exercise in males and females. We hypothesized that blood pressure responses during post‐exercise circulatory arrest would be significantly attenuated with capsaicin. Specifically, we posited that the male metaboreflex responses would be attenuated and female responses would not, given EPR sex differences. Our second hypothesis was that capsaicin application would improve microvascular responsiveness similarly in both men and women, given previous reports indicating that the menstrual phase has minimal effect on these responses (Mattu et al., 2020).

## 2 METHODS

### 2.1 Participants

Twenty healthy college‐aged males (9) and females (11) [age 21 ± 1 years (mean ± SD); height 171 ± 10 cm; mass 71 ± 1 kg; body mass index 25 ± 3 kg/m^2^] volunteered to participate in the current investigation (Table 1). Criteria used for volunteers were nonsmokers, cardiovascular disease (CVD) free, and cardiac medication free. Female contraceptive use was not controlled, but was tested during self‐reported early follicular (<1 week post menstruation) or late luteal phases (1‐week prior to menstruation). All experimental procedures were approved by the Institutional Review Board of the University of Wisconsin‐La Crosse and conformed to the standards set forth by the Declaration of Helsinki. Written informed consent and health history screening for overt diseases (e.g., cardiovascular, metabolic, and renal) took place before data collection. All data testing was completed in a temperature‐controlled laboratory (20–22°C).

**TABLE 1:** Displays participant baseline characteristics, forearm tissue thickness (FTT) (*n* = 20 [11 F]).

*n*	20 (11 F)
Age, year	21 ± 1
BMI, kg/m^2^	25 ± 3
Height, cm	171 ± 10
Mass, kg	72 ± 1
MVC, kg	37 ± 9
Tissue thickness, cm	0.3 ± 0.1

### 2.2 Experimental protocol

**FIGURE 1:**
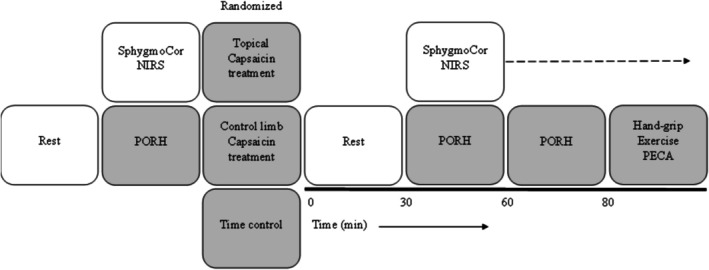
Displays the study overview.

The current investigation was a semi‐randomized crossover design (females testing was counter‐balanced based on self‐reported menstrual phase), separated by a 48‐h washout period (Figure 1). Participants were randomly assigned to either capsaicin applied to the exercising arm (CAP), capsaicin applied to the non‐exercising arm (SYSTEMIC CONTROL), or capsaicin‐free (TIME CONTROL). Female participants self‐reporting menstrual cycle performed an additional CAP treatment during their late luteal phase to study the effect of capsaicin in a high sex hormone phase. All participants were instructed to abstain from alcohol, caffeine, and exercising for at least 12 h and be at least 4 h fasted prior to laboratory visits. Testing was completed at varying times throughout the day, but always within 1 h of each participant's repeated visit.

### 2.3 Familiarization

During the first lab visit, the height and mass of each participant were taken. Next, participants were instructed to lay supine to obtain maximal voluntary contraction (MVC) of the right forearm by extending their right arm at heart level and to familiarize them with the protocol. Participants were instructed to exert maximal force on a handgrip dynamometer for 2 s with 1 min of rest intervals in triplicate. MVC was calculated based on the average of the two highest values and was used to calculate the percentage for rhythmic exercise testing: 30% MVC. While isometric exercise may lead to greater metabolite buildup, we chose rhythmic exercise to enhance ecological validity and better reflect real‐world relevance for our target population, and the >20 mmHg change in MAP suggests a sufficient metabolically driven pressor response.

Forearm flexors were then identified by a trained researcher during handgrip contraction via palpation. The identified area of the volar forearm was then marked with a surgical marker, and tissue thickness was taken with an ultrasound probe placed over the skin. Following confirmation of appropriate subcutaneous adipose thickness, a near‐infrared spectrometer was placed over the identified area of skin for familiarization. Rhythmic handgrip exercise was then performed on a custom‐built hand‐grip dynamometer in the right arm at a rate of 20 contractions per min at 30% MVC for 3 min. 10 s prior to ending exercise, post‐exercise circulatory arrest was initiated to familiarize participants. Circulatory arrest was performed with the rapid inflation Hokanson cuff to 250 mmHg.

### 2.4 Experimental testing

Testing was completed at varying times throughout the day (e.g., 8:00 am–11:00 am and 1:00 pm–4:00 pm), but always within 1 h of each participant's repeated visit. For every visit following familiarization, participants reported to the lab and a 20 min supine rest began. Following rest, measurement of blood pressure (BP) in duplicate was recorded using the SphygmoCor XCEL (Atcor Medical, Sydney, AU). Next, a near‐infrared spectroscopy (NIRS) post occlusive reactive hyperemia (PORH) test was performed. Participants were then randomly assigned to one of three treatments: capsaicin applied to the exercising arm (CAP), capsaicin applied to the non‐exercising arm (SYSTEMIC CONTROL), or capsaicin‐free (TIME CONTROL). In treatments where capsaicin was applied, 1 g of 0.1% capsaicin topical cream from a single batch (Capzasin HP, Chattem Incorporated, Chattanooga, Tennessee) (LOT#24‐05363) was applied to the volar forearm region, like previous reports (Vianna et al., 2018). If CAP was selected, the NIRS device was removed and the topical capsaicin cream was applied before replacing the device in the same spot. Participants rested before a NIRS PORH test was performed at a time point 30 min post treatment (T30) and repeated 60 min post treatment (T60). Hand‐grip exercise only occurred after 10 min of rest from the end of T60 NIRS PORH measurements and concluded with post exercise circulatory arrest (PECA) (Figure 1).

## 3 EXPERIMENTAL MEASURES

### 3.1 Cardiovascular measures

Brachial derived mean arterial pressure (MAP) and heart rate (HR) were assessed using an automated oscillometric device (SphygmoCor XCEL: Atcor Medical, Sydney, AU) and averaged from at least two readings with <5 mmHg difference (Table 2) (Kang et al., 2022). The brachial cuff was placed on the participants' left upper arm. MAP and HR were taken in duplicate at baseline, and time points T30, T60, and during PECA. To account for time to peak cuff pressure during PECA, the cuff inflation was initiated at 1 min 30 s into PECA, and we took a single BP measurement. For all measurements, participants were constantly reminded to keep their arm relaxed at their side and to refrain from any movement during the inflation and waveform measurement periods.

**TABLE 2:** Displays resting blood pressure and heart rate data between capsaicin (CAP), systemic control (SC), and time control (TC) (*n* = 20 [11 F]).

	CAP		Systemic control		Time control		Two‐way repeated measures ANOVA		
	Males	Females	Males	Females	Males	Females	Sex	Treatment	Interaction
Systolic blood pressure, mmHg	105 ± 33	99 ± 8	93 ± 7	99 ± 8	104 ± 12	100 ± 10	0.185	0.785	0.819
Diastolic blood pressure, mmHg	67 ± 21	69 ± 7	60 ± 5	71 ± 9	67 ± 7	70 ± 9	0.322	0.742	0.438
Mean arterial blood pressure, mmHg	81 ± 25	81 ± 9	71 ± 5	83 ± 9	80 ± 8	83 ± 10	0.575	0.794	0.561
Heart rate, bpm	59 ± 19	65 ± 11	53 ± 10	69 ± 13	58 ± 9	70 ± 9	0.042*	0.39	0.284

*Note*: Data were run with two‐way repeated measures analysis of variance. Data are represented as means ± standard deviation.

* Indicates *p* value <0.05.

### 3.2 Near‐infrared spectroscopy

Microvascular hemoglobin + myoglobin [Heme] was measured with a continuous wave multidistance NIRS probe (PortaMon, Artenis) that was placed longitudinally over the flexor digitorum muscle belly, volar region, of the forearm. In brief, the NIRS probe consists of a detector fiber bundle and three light‐emitting diodes (LEDs), and operates at wavelengths of 690 and 830 nm (source detector distance 2.5–4.0 cm) with a maximal penetration depth of 2.0 cm. An ultrasound was used to quantify the area of NIRS interrogation for adipose thickness before placement. The NIRS device allows for relative changes in oxygenated (oxy‐[Heme]), deoxygenated (deoxy‐[Heme]), and the calculated sum, total (total‐[Heme]). The use of [Heme] is preferred as the NIRS cannot distinguish between hemoglobin and myoglobin (Barstow, 2019). The present work uses muscle tissue saturation, which is calculated as oxy‐[Heme] divided by total‐[Heme]. The NIRS probe was allowed to run for at least 2 min before the start of data collection. Identification of the muscle belly was carried out by a single experienced investigator palpating during muscular contraction, being removed and placed back for treatment application. The NIRS data were collected throughout testing at 10 Hz and exported at 1 Hz. The NIRS parameters were calculated as follows: (1) baseline percent saturation was calculated as an average over the minute before cuff inflation; (2) desaturation rate was quantified as the downward slope from 10 to 60 s following cuff inflation; (3) tissue resaturation rate was quantified as the upward slope of the 10 s window following cuff release; (4) nadir was calculated as the minimum value during occlusion; (5) peak was calculated as the maximal value after cuff release; (6) AUC was calculated using the trapezoid rule during occlusion after cuff deflation (Barstow, 2019; McLay, Fontana, et al., 2016; McLay, Nederveen, et al., 2016).

## 4 STATISTICS

Reviewers were blinded to treatment conditions during all analyses. All data was analyzed with commercially available statistical software (Sigmaplot; Version 14.5, Systat software, San Jose). MAP and HR differences from baseline (∆MAP/∆HR) were initially analyzed with a two‐way (Sex × Treatment) repeated measures analysis of variance (ANOVA) without significance. Accordingly, we then analyzed data with a one‐way repeated ANOVA comparing treatments across both sexes. Microvascular NIRS data were analyzed with a three‐way (Sex × Treatment × Time) repeated measures analysis of variance ANOVA without significance. Accordingly, we then analyzed data with a two‐way (Treatment × Time) repeated measures ANOVA with Student–Newman–Keuls post hoc for pairwise comparison if significant interactions were found. Menstrual cycles were compared with a two‐way ANOVA (Phase × Time). The level of significance was set at (*p* < 0.05). All data are presented as means ± standard deviation.

## 5 RESULTS

### 5.1 Mean arterial pressure and heart rate responses

**FIGURE 2:**
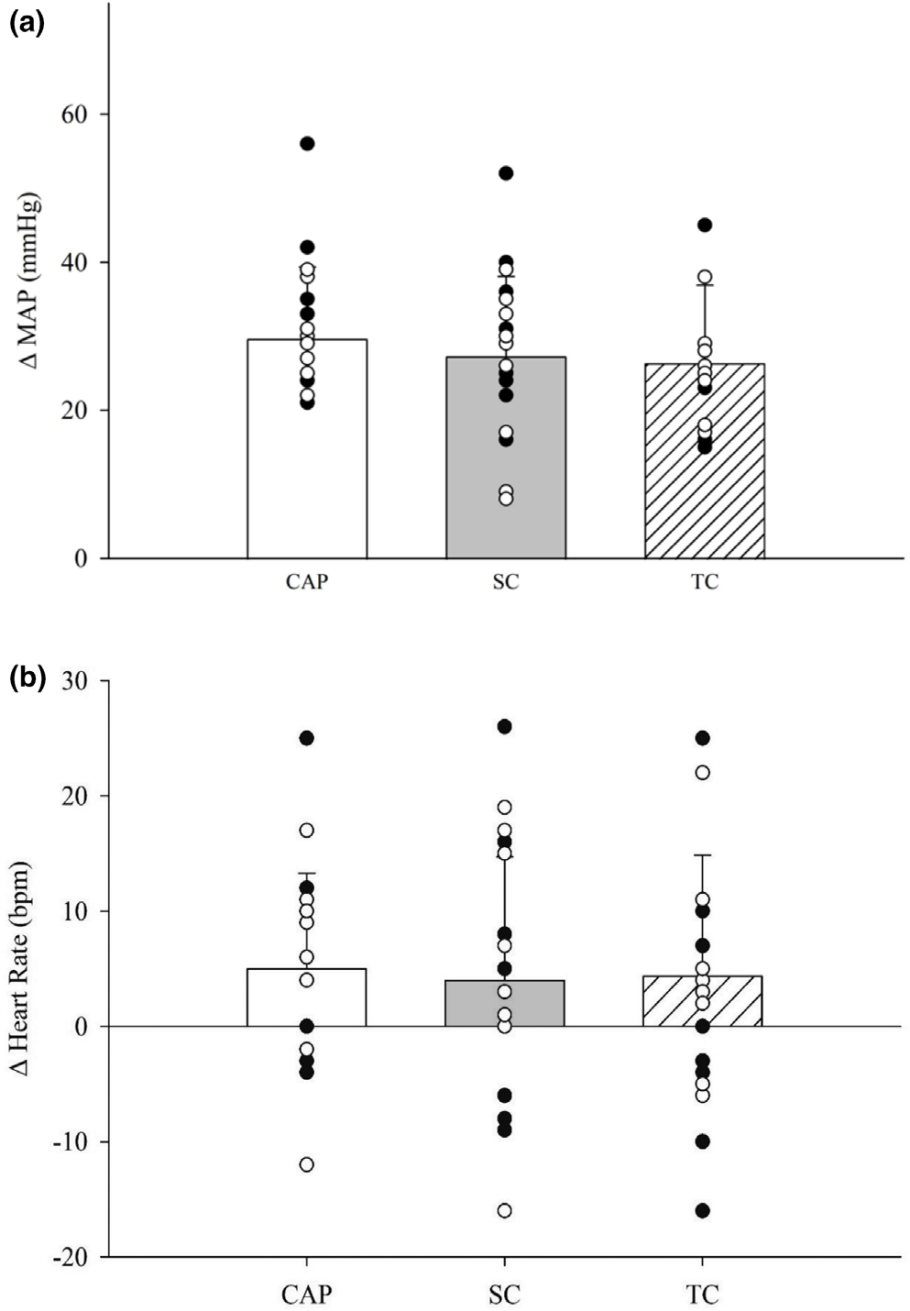
(a) Displays changes in mean arterial pressure from baseline (ΔMAP) between capsaicin (CAP), systemic control (SC), and time control (TC) during post exercise circulatory arrest in males (black) and females (white), and (b) Changes in heart rate from baseline (ΔHR) between CAP (*n* = 20), SC (*n* = 20), and TC (*n* = 20) during post exercise circulatory arrest in males and females (*n* = 20 [11 F]). Data were run with one‐way analysis of variance. Data are means ± standard deviations.

Two‐way ANOVA analysis on resting timepoints of T30 and T60 revealed that MAP changes from baseline (∆MAP) for treatment (*p* = 0.32) and time (*p* = 0.45) were not statistically different (Table 2). Similarly, our analysis revealed that HR changes from baseline (∆HR) were not different for treatment (*p* = 0.79) or time (*p* = 0.56) (Table 3). To examine ∆MAP during PECA, a one‐way ANOVA revealed a moderate effect size of capsaicin (Cohen's *d* = 0.5) yet, no significant difference for treatment (*p* = 0.31) (Figure 2a). Moreover, no changes in ∆HR between treatments during PECA were shown (*p* = 0.73) with a low effect size of capsaicin (Cohen's *d* = 0.1) (Figure 2b). When considering the menstrual cycle, the luteal phase had no impact on ∆MAP (*p* = 0.18) or ∆HR (*p* = 0.96) during PECA when compared to the early follicular phase (Table S1).

**TABLE 3:** Displays changes in mean arterial pressure from baseline (ΔMAP) and changes in heart rate from baseline (ΔHR) between capsaicin (CAP), systemic control (SC), and time control (TC) at time points 30‐ and 60 min (*n* = 20 [11 F]).

	CAP		Systemic control		Time control		Two‐way repeated measures ANOVA		
	Males	Females	Males	Females	Males	Females	Sex	Treatment	Interaction
T30									
ΔMAP, mmHg	3 ± 6	1 ± 3	2 ± 5	1 ± 5	0 ± 6	1 ± 5	0.118	0.456	0.489
ΔHR, bpm	3 ± 4	1 ± 7	3 ± 4	5 ± 5	3 ± 8	3 ± 4	0.518	0.352	0.771
T60									
ΔMAP, mmHg	4 ± 5	1 ± 5	5 ± 8	2 ± 3	1 ± 6	1 ± 4	0.25	0.324	0.198
ΔHR, bpm	2 ± 2	2 ± 5	2 ± 3	8 ± 5	2 ± 6	4 ± 5	0.77	0.573	0.971

*Note*: Data were run with 2‐way repeated measures analysis of variance. Data are represented as means ± standard deviation.

### 5.2 NIRS‐derived responses

**FIGURE 3:**
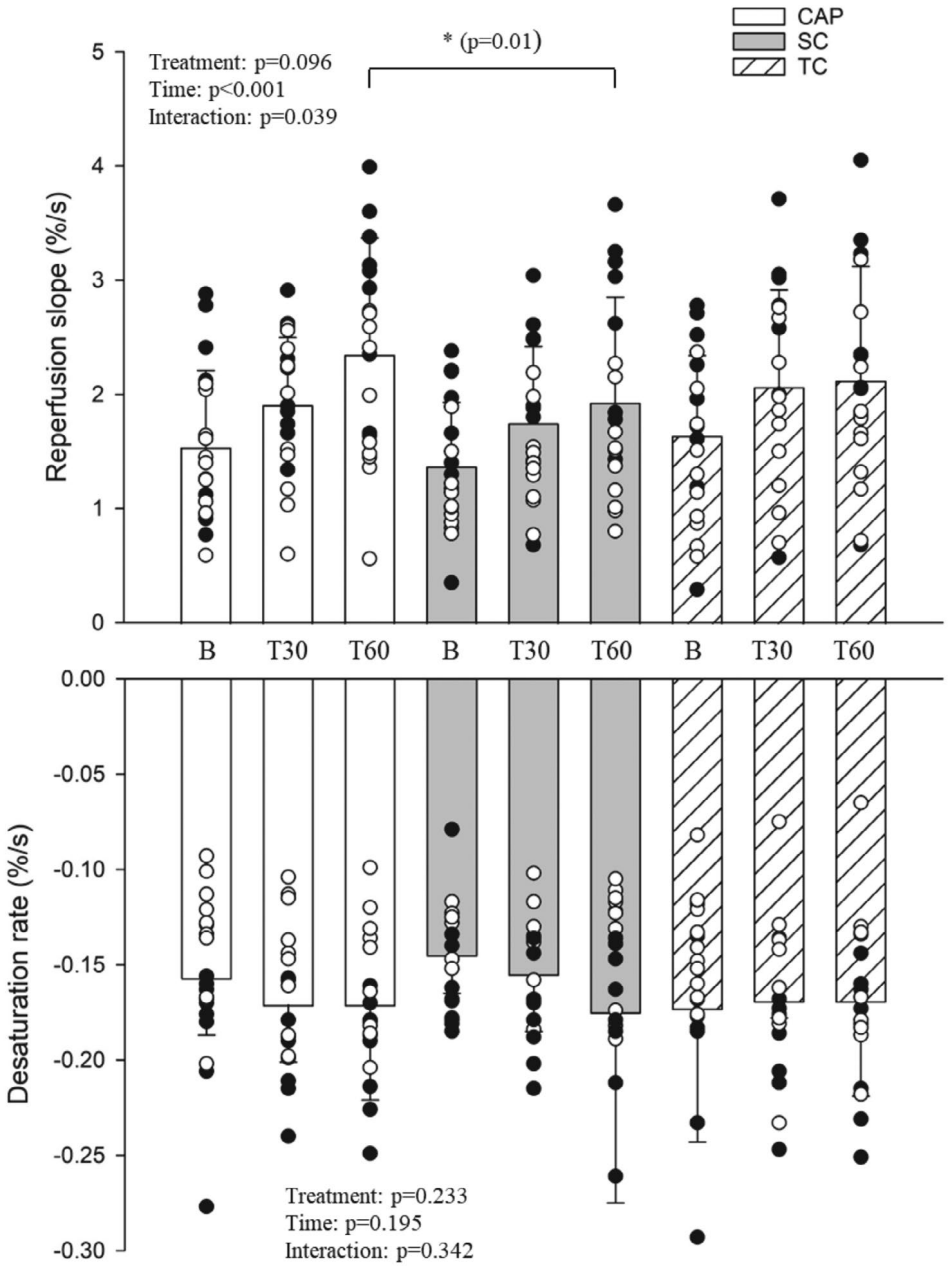
Displays near infrared spectroscopy derived microvascular reperfusion slope responses to capsaicin (CAP) (*n* = 20), systemic control (SC) (*n* = 20), and time control (TC) (*n* = 20) treatments in males (black) and females (white). Data were run with two‐way repeated measures analysis of variance. Data are means ± standard deviation. *Indicates *p* value <0.05.

Baseline measurement for NIRS microvascular desaturation, baseline % saturation, occlusion area under the curve (AUC), or nadir during occlusion were not different between treatments (Table 4). Microvascular responsiveness (NIRS‐derived tissue reperfusion rate) was increased 60 min after capsaicin placement (CAP: 2.35 ± 1.02%/s) relative to the non‐exercise arm (SYSTEMIC CONTROL: 1.93 ± 0.92%/s), but not TIME CONTROL: 2.12 ± 1.0%/s (*p* = 0.01) (Figure 3). When female hormonal phase was compared between time points with capsaicin on the exercising arm, no interaction occurred, but an effect of time for changes in MAP and reperfusion rates was present (*p* > 0.05, Table S1 https://doi.org/10.6084/m9.figshare.29365838.v1).

**TABLE 4:** Displays resting near‐infrared spectroscopy derived microvascular measurements (Baseline, Nadir, Peak, Recovery, Slope 1, Area Under Curve) between capsaicin (CAP), systemic control (SC), and time control (TC) (*n* = 20 [11 F]).

	CAP	Systemic control	Time control	Two‐way repeated measures ANOVA		
				Time	Treatment	Interaction
Resting						
Baseline, %	69.5 ± 2.9	67.7 ± 3.3	70.0 ± 2.9	0.10	0.018	0.259
NADIR, %	29.6 ± 9.0	30.1 ± 8.0	30.1 ± 9.3	0.092	0.541	0.211
Peak, %	81.3 ± 3.1	77.4 ± 12.0	80.4 ± 3.8	0.150	0.178	0.118
Recovery, %	70.9 ± 10.4	67.5 ± 11.1	70.9 ± 4.4	0.223	0.386	0.782
AUC, AU	21316.4 ± 17382.8	19162.5 ± 12853.1	22220.5 ± 20540.7	0.360	0.361	0.460
T30						
Baseline, %	68.7 ± 4.0	68.4 ± 3.3	70.5 ± 2.7			
NADIR, %	26.8 ± 11.0	29.8 ± 7.8	29.8 ± 9.6			
Peak, %	80.6 ± 3.3	80.6 ± 2.8	82.0 ± 2.8			
Recovery, %	70.5 ± 4.0	70.2 ± 6.0	71.9 ± 4.0			
AUC, AU	20103.2 ± 12198.5	19554.6 ± 13007.0	29742.9 ± 15567.0			
T60						
Baseline, %	70.3 ± 3.4	68.9 ± 3.2	70.4 ± 2.6			
NADIR, %	28.2 ± 10.9	29.3 ± 8.2	30.2 ± 9.6			
Peak, %	80.9 ± 3.1	80.9 ± 2.8	82.0 ± 2.6			
Recovery, %	71.2 ± 3.0	70.4 ± 6.6	72.1 ± 3.8			
AUC, AU	20809.1 ± 14142.4	17737.3 ± 6764.0	21170.7 ± 10418.6			

*Note*: Data are represented as means ± standard deviation. Data were run with two‐way repeated measures analysis of variance.

## 6 DISCUSSION

This study is the first to determine the effects of topical capsaicin cream on the metaboreflex and NIRS derived microvascular responses prior to and during rhythmic handgrip exercise in young healthy males and females. We hypothesized that capsaicin would attenuate male, but not female, blood pressure responses during PECA given the blunted female metaboreflex (Gonzales et al., 2007; Hunter & Enoka, 2001; Limberg et al., 2010; Ogawa et al., 1992; Parker et al., 2007). We also hypothesized that capsaicin application would increase microvascular responsiveness in both sexes. Our results suggest that topical capsaicin did not attenuate the metaboreflex during post‐exercise circulatory arrest, but did increase microvascular responsiveness 60‐min post application relative to the systemic control condition.

Topical capsaicin cream had no impact on the change in mean arterial pressure from baseline (ΔMAP) to post‐exercise circulatory occlusion (PECA). This finding contrasts with previous work by Dawson et al., 2004, who recorded attenuated pressor responses in both sexes with capsaicin cream 50 min after application (Dawson et al., 2004). However, these investigators did not attempt to isolate the metaboreflex through PECA and utilized a capsaicin solution containing methyl salicylate, a non‐steroidal anti‐inflammatory drug that may alter metabolically sensitive afferents. In a recent investigation, ΔMAP was reduced at 30‐ and 60‐min following capsaicin application but did not include females (Vianna et al., 2018). While aiming to extend our understanding of biological sex's role in capsaicin's attenuation of pressor responses, we have addressed no ability of capsaicin to impact metaboreflex responses in either sex. It is possible that differences in experimental design impair the ability to reproduce previous results of attenuated ΔMAP responses to PECA. Future studies should continue to control for capsaicin cream dose response and timing of application to testing, as both variables are likely to contribute to metaboreflex MAP responses.

The female metaboreflex responses herein suggest no impact of self‐reported menstrual phase (Table S1). We collected data from young healthy females at their self‐reported low hormone, early follicular, and high hormone, late luteal phases. This finding is in line with previous investigations that studied the metaboreflex responses at high and low female sex hormone concentration and showed no effect of female menstrual phase (Jarvis et al., 2011). Acute alterations in menstrual phase have been suggested to have a limited impact on EPR responses in premenopausal women (Jarvis et al., 2011; Smith et al., 2019). Interestingly, when comparing male and female PECA responses, our data do not illustrate the ubiquitous blunted female pressor response that so many other investigators have identified in various protocols (Ettinger et al., 1996; Hunter & Enoka, 2001; Jarvis et al., 2011; Limberg et al., 2010; Minahan et al., 2018; Ogawa et al., 1992; Parker et al., 2007). It is currently unknown if age, activity, or other factors influence differences between investigations. As such, the need to standardize the female menstrual cycle may not be necessary, although future work confirming our results with more rigorous hormone profile measures is needed.

### 6.1 Topical capsaicin and microvascular responsiveness

Our findings provide partial support for the use of topical capsaicin to influence microvascular responsiveness. Capsaicin applied to the exercising forearm increased microvascular responsiveness 60 min post‐application relative to the systemic control condition; however, no significant difference was observed when compared to the capsaicin‐free condition. This may suggest that when capsaicin is applied to non‐exercising muscle, there is an attenuated effect. Considering recent findings that oral capsaicin ingestion reduced microvascular responsiveness in females (Zaleski et al., 2023), our results highlight the complexity of capsaicin's effects and the importance of administration route and target tissue. This study is the first to examine forearm microvascular responses across multiple timepoints following topical capsaicin application, providing insight into its temporal effects on skeletal muscle perfusion. Capsaicin has been previously shown to exert vasodilatory effects via TRPV1 receptor activation in both human and animal models (Lv et al., 2015; Randhawa & Jaggi, 2017; Yang et al., 2010), and we speculate that similar mechanisms may underlie the observed changes in reperfusion rate. Nonetheless, differences in study design and delivery method underscore the need for further research. Finally, consistent with changes in ΔMAP, we observed no apparent effect of self‐reported menstrual cycle phase on microvascular responses (Mattu et al., 2020).

### 6.2 Limitations

It is important to note the limitations of the current study. The current study did not directly measure and confirm self‐reported female menstrual cycle. Future studies investigating sex differences and hormone influence should directly measure female estrogen and progesterone levels as well as body temperature to confirm relatively low and high hormone phases. Additionally, we did not control for contraceptive use. It could be suggested that skin blood flow impacted the microvascular responses at rest shown herein. However, various NIRS parameters (e.g., baseline tissue saturation, desaturation slope, and AUC during occlusion) were similar between treatments and time points, confirming minimal influence of skin blood flow. Further, the adipose tissue depth was (0.3 ± 0.1 cm) and with a NIRS penetration depth up to 1.0 cm, it is unlikely to be impacted. TRPV‐1 receptors agonized by capsaicin play a significant role in nociception, and it is important to note that we did not measure pain perception or compare its influence on the autonomic cardiovascular responses. Valuable insights could be provided from future studies incorporating a visual analog scale of pain perception and analyzing the impact on cardiovascular measures. The current work used rhythmic handgrip exercise over isometric and is more difficult to compare to previous studies, yet the delta changes were in line with what is commonly shown in the literature.

## 7 CONCLUSIONS

In the present study, we found that topical capsaicin cream had no effect on metaboreflex responses during PECA, but it did increase microvascular reperfusion rate relative to the systemic control condition. Although a significant knowledge gap remains regarding the topical effects of capsaicin on exercise‐induced and female‐specific pressor responses in humans, future studies incorporating direct measurements of hormonal fluctuations could enhance our understanding of the EPR. Additionally, further research is warranted to examine the influence of post‐treatment timing to clarify the extent and reproducibility of capsaicin's effects.

## AUTHOR CONTRIBUTIONS

J.T.C. and N.D.C. conceived and designed the research; N.D.C., A. E. W., and J.T.C. performed experiments; N.D.C. and J.T.C. drafted the manuscript and provided editing. J.T.C. approved the final version of the manuscript.

## FUNDING INFORMATION

This work was supported by the University of Wisconsin‐La Crosse Graduate Research funding.

## CONFLICT OF INTEREST STATEMENT

No conflicts of interest, financial or otherwise, are declared by the authors.

## Supporting information


Table S1.

## Data Availability

Data will be made available upon reasonable request.
