# Supplementary material for: The impact of topical capsaicin application on the muscle metaboreflex and microvascular responsiveness
Source: Physiol Rep. 2025 Aug 9;13(15):e70496. doi: 10.14814/phy2.70496 (PMC12334846; doi:10.14814/phy2.70496)
Supplement: Supplementary file 1 — Table S1. [file PHY2-13-e70496-s001.docx]

| **Table S1** |  | | | | | | | | | | |  |  |  |  |  |  |  |
| --- | --- | --- | --- | --- | --- | --- | --- | --- | --- | --- | --- | --- | --- | --- | --- | --- | --- | --- |
|  | Pre-Treatment | | |  | T30 | | |  | T60 | | |  | PECA | | | Two-way ANOVA | | |
| ***Cardiovascular*** | Early Follicular (n = 10) | | | | | | | | | | |  |  |  |  | Phase | Time | Interaction |
| Systolic blood pressure, mmHg | 99 | ± | 8 |  |  |  |  |  |  |  |  |  |  |  |  |  |  |  |
| Diastolic blood pressure, mmHg | 69 | ± | 7 |  |  |  |  |  |  |  |  |  |  |  |  |  |  |  |
| ∆ Mean arterial pressure, mmHg |  |  |  |  | 0 | ± | 4 |  | 1 | ± | 5 |  | 29 | ± | 6 | 0.8 | **<.001*** | 0.44 |
| ∆ Heart rate, bpm |  |  |  |  | -1 | ± | 7 |  | -2 | ± | 5 |  | 5 | ± | 8 | 0.17 | **<.001*** | 0.65 |
|  | Late Luteal (n = 8) | | | | | | | | | | |  |  |  |  |  |  |  |
| Systolic blood pressure, mmHg | 99 | ± | 11 |  |  |  |  |  |  |  |  |  |  |  |  |  |  |  |
| Diastolic blood pressure, mmHg | 69 | ± | 10 |  |  |  |  |  |  |  |  |  |  |  |  |  |  |  |
| ∆ Mean arterial pressure, mmHg |  |  |  |  | 2 | ± | 6 |  | 1 | ± | 6 |  | 27 | ± | 8 |  |  |  |
| ∆ Heart rate, bpm |  |  |  |  | -5 | ± | 7 |  | -6 | ± | 7 |  | 4 | ± | 8 |  |  |  |
|  | Pre-Treatment | | |  | T30 | | |  | T60 | | |  | HGE | | |  |  |  |
| ***Near infrared spectroscopy*** | Early Follicular (n = 10) | | | | | | | | | | |  |  |  |  | Phase | Time | Interaction |
| Baseline | 70.5 | ± | 3.5 |  | 71.0 | ± | 3.3 |  | 70.7 | ± | 3.1 |  | 70.1 | ± | 3.2 | 0.32 | 0.55 | 0.7 |
| Nadir % | 34.5 | ± | 7.3 |  | 34.6 | ± | 11.4 |  | 34.9 | ± | 10.9 |  | 33.4 | ± | 11.4 | 0.85 | 0.99 | 0.99 |
| Peak % | 81.1 | ± | 2.6 |  | 82.2 | ± | 2.9 |  | 82.0 | ± | 3.1 |  | 76.9 | ± | 3.7 | 0.07 | 0.03 | 0.26 |
| Recovery % | 71.5 | ± | 4.1 |  | 72.2 | ± | 5.4 |  | 72.4 | ± | 4.8 |  | 72.4 | ± | 4.7 | 0.15 | 0.92 | 0.77 |
| Slope 1 %/s | -0.1 | ± | 0.0 |  | -0.2 | ± | 0.0 |  | -0.2 | ± | 0.0 |  | -0.2 | ± | 0.1 | 0.59 | 0.21 | 0.8 |
| Slope 2 %/s | 1.4 | ± | 0.5 |  | 1.8 | ± | 0.7 |  | 1.8 | ± | 0.7 |  | 2.4 | ± | 1.3 | 0.44 | **0.002*** | 0.74 |
| AUC | 17105.7 | ± | 3408.2 |  | 20828.8 | ± | 9672.9 |  | 19064.8 | ± | 10138.6 |  | 12614.3 | ± | 632.1 | 0.10 | 0.62 | 0.75 |
|  | Late Luteal (n = 8) | | | | | | | | | | |  |  |  |  |  |  |  |
| Baseline | 68.8 | ± | 3.0 |  | 70.0 | ± | 3.3 |  | 70.8 | ± | 3.3 |  | 70.8 | ± | 3.6 |  |  |  |
| Nadir % | 35.3 | ± | 6.4 |  | 35.1 | ± | 6.7 |  | 35.0 | ± | 6.3 |  | 36.5 | ± | 5.3 |  |  |  |
| Peak % | 77.8 | ± | 4.5 |  | 81.4 | ± | 2.6 |  | 81.7 | ± | 1.9 |  | 78.4 | ± | 2.6 |  |  |  |
| Recovery % | 70.4 | ± | 9.2 |  | 67.8 | ± | 13.5 |  | 67.3 | ± | 12.6 |  | 69.9 | ± | 9.2 |  |  |  |
| Slope 1 %/s | -0.1 | ± | 0.0 |  | -0.2 | ± | 0.0 |  | -0.1 | ± | 0.0 |  | -0.2 | ± | 0.1 |  |  |  |
| Slope 2 %/s | 1.1 | ± | 0.6 |  | 1.7 | ± | 0.8 |  | 1.8 | ± | 0.7 |  | 2.3 | ± | 0.6 |  |  |  |
| AUC | 14013.6 | ± | 2196.7 |  | 14487.9 | ± | 2279.8 |  | 14389.1 | ± | 1798.2 |  | 15627.0 | ± | 8341.1 |  |  |  |

Female cardiovascular and near-infrared spectroscopy responses
